# Supplementary material for: Fine-tuning protein language models boosts predictions across diverse tasks
Source: Nat Commun. 2024 Aug 28;15:7407. doi: 10.1038/s41467-024-51844-2 (PMC11358375; doi:10.1038/s41467-024-51844-2)
Supplement: Supplementary file 3 — Reporting Summary [file 41467_2024_51844_MOESM3_ESM.pdf]

nature portfolio

Corresponding author(s): Robert Schmirler

Last updated by author(s): Jul 30, 2024

Reporting Summary

Nature Portfolio wishes to improve the reproducibility of the work that we publish. This form provides structure for consistency and transparency in reporting. For further information on Nature Portfolio policies, see our Editorial Policies and the Editorial Policy Checklist.

Statistics

For all statistical analyses, confirm that the following items are present in the figure legend, table legend, main text, or Methods section.

n/a	Confirmed	
☐	☒	The exact sample size (n) for each experimental group/condition, given as a discrete number and unit of measurement
☐	☒	A statement on whether measurements were taken from distinct samples or whether the same sample was measured repeatedly
☒	☐	The statistical test(s) used AND whether they are one- or two-sided<br>*Only common tests should be described solely by name; describe more complex techniques in the Methods section.*
☒	☐	A description of all covariates tested
☒	☐	A description of any assumptions or corrections, such as tests of normality and adjustment for multiple comparisons
☐	☒	A full description of the statistical parameters including central tendency (e.g. means) or other basic estimates (e.g. regression coefficient) AND variation (e.g. standard deviation) or associated estimates of uncertainty (e.g. confidence intervals)
☒	☐	For null hypothesis testing, the test statistic (e.g. F, t, r) with confidence intervals, effect sizes, degrees of freedom and P value noted<br>*Give P values as exact values whenever suitable.*
☒	☐	For Bayesian analysis, information on the choice of priors and Markov chain Monte Carlo settings
☒	☐	For hierarchical and complex designs, identification of the appropriate level for tests and full reporting of outcomes
☒	☐	Estimates of effect sizes (e.g. Cohen's d, Pearson's r), indicating how they were calculated

Our web collection on statistics for biologists contains articles on many of the points above.

Software and code

Policy information about availability of computer code

Data collection	No software was used to collect data
Data analysis	All Software used during this study is described in code availability section:<br>We made the notebooks for fine-tuning all our models (Table 1) for per-protein and per-residue tasks available at https://github.com/RSchmirler/data-repo_plm-finetune-eval (DOI: 10.5281/zenodo.12770310). By default, these notebooks use LoRA fine-tuning; full-model fine-tuning is optional. To create prediction methods based on pre-trained pLM embeddings, we provided two additional notebooks (one to generate embeddings, and another to train predictors) with sample data in the same repository.<br>RFA v1.0.0 R scripts are available from https://github.com/whatdoidohaha/RFA.

For manuscripts utilizing custom algorithms or software that are central to the research but not yet described in published literature, software must be made available to editors and reviewers. We strongly encourage code deposition in a community repository (e.g. GitHub). See the Nature Portfolio guidelines for submitting code & software for further information.

Data

Policy information about availability of data

All manuscripts must include a data availability statement. This statement should provide the following information, where applicable:

- Accession codes, unique identifiers, or web links for publicly available datasets
- A description of any restrictions on data availability
- For clinical datasets or third party data, please ensure that the statement adheres to our policy

All data sets analyzed are freely available through the original sources:
GFP and stability: https://github.com/songlab-cal/tape
AAV, GB1, meltome and secondary structure: https://github.com/J-SNACKKB/FLIP}
Sub-cellular location: https://github.com/HannesStark/protein-localization
Disorder: https://github.com/DagmarIlz/SETH
Easing access, we re-packaged all data at https://github.com/RSchmirler/data-repo_plm-finetune-eval (DOI: 10.5281/zenodo.12770310). When using those data, please quote and consult the authors of the original data sets. All data generated for this study and source data to generate figures and tables is also available in this repository.
Source data are provided with this paper.

Research involving human participants, their data, or biological material

Policy information about studies with human participants or human data. See also policy information about sex, gender (identity/presentation), and sexual orientation and race, ethnicity and racism.

Reporting on sex and gender	na
Reporting on race, ethnicity, or other socially relevant groupings	na
Population characteristics	na
Recruitment	na
Ethics oversight	na

Note that full information on the approval of the study protocol must also be provided in the manuscript.

Field-specific reporting

Please select the one below that is the best fit for your research. If you are not sure, read the appropriate sections before making your selection.

☒ Life sciences ☐ Behavioural & social sciences ☐ Ecological, evolutionary & environmental sciences

For a reference copy of the document with all sections, see nature.com/documents/nr-reporting-summary-flat.pdf

Life sciences study design

All studies must disclose on these points even when the disclosure is negative.

Sample size	No sample size calculation was performed, instead sample sizes (reruns of the same experiment with different random state) was chosen based on available computational ressources.<br>For more expensive finetuning experiments we chose 3 reruns, for the cheaper embedding based predictors we chose 5.<br>The number of samples is given with the individual results.
Data exclusions	During our research we excluded a single training run (Finetuning ESM2 150M on the AAV dataset, seed 98) which was a clear outlier. For details check Supplementary Online Material Table S2.
Replication	Replication of computational results is made possible by defining random states (seed). These random seeds are given in the detailed results (Table S1 and S2). The code and data provided by us will allow complete reproduction of our results, in addition results for each individual model training run are provided with the source data.
Randomization	Since random data splits lead to a large overestimation of predictor performance, we used dataset splits from previous work specifically designed to test for generalization.
Blinding	There are no group allocations in this study.

Reporting for specific materials, systems and methods

We require information from authors about some types of materials, experimental systems and methods used in many studies. Here, indicate whether each material, system or method listed is relevant to your study. If you are not sure if a list item applies to your research, read the appropriate section before selecting a response.

Materials & experimental systems

n/a	Involved in the study
☒	☐ Antibodies
☒	☐ Eukaryotic cell lines
☒	☐ Palaeontology and archaeology
☒	☐ Animals and other organisms
☒	☐ Clinical data
☒	☐ Dual use research of concern
☒	☐ Plants

Methods

n/a	Involved in the study
☒	☐ ChIP-seq
☒	☐ Flow cytometry
☒	☐ MRI-based neuroimaging

Plants

Seed stocks	na
Novel plant genotypes	na
Authentication	na
